# Analysis of Clinical Characteristics of Pertussis in Children Aged 0–6 Years in Tertiary and Pediatric Hospitals of Jiangsu Province from January to May 2024

**DOI:** 10.3390/vaccines14090806

**Published:** 2026-09-14

**Authors:** Yan Xu, Mei Li, Qiang Chen, Chengmei Jia, Lingyan Tang, Yuanbao Liu, Zhiguo Wang, Xiang Huo

**Affiliations:** 1 Department of Immunization Program, Jiangsu Provincial Center for Disease Control and Prevention, Nanjing 210009, China; xuyan1369@163.com (Y.X.);; 2 Department of Program on Immunization, Wujin District Center for Disease Control and Prevention, Changzhou 213164, China

**Keywords:** pertussis, clinical characteristics, influencing factors, hospitalization

## Abstract

**Objective:** To analyze the clinical manifestations and hospitalization-related factors among children with pertussis, and to provide scientific support for pertussis prevention, control, clinical diagnosis, and treatment. **Methods:** From January through May 2024, children aged 0–6 years with a confirmed diagnosis of pertussis at tertiary hospitals and children’s hospitals in Jiangsu Province were enrolled in this investigation. Descriptive statistical analysis was performed to explore the clinical characteristics of the children, with stratification by age, immunization status, time from onset to diagnosis, and visit type. Logistic regression analysis was applied to identify factors associated with hospitalization. **Results:** In total, 3361 children were recruited, with the 6-year age group accounting for the highest proportion (35.4%). Severe symptoms such as apnea and cyanosis were more common in infants aged 0–5 months. Children who received four doses of the pertussis vaccine had significantly lower rates of paroxysmal cough, vomiting, cyanosis and apnea than unvaccinated children (*p* < 0.001). Delayed diagnosis (≥15 days after onset) was found in 26.89% of children, who had a higher prevalence of vomiting and cyanosis. The prevalence of all clinical symptoms was higher in hospitalized children than in outpatients. Age 0–2 months, delayed diagnosis, cyanosis, paroxysmal cough, and fever were positively associated with hospitalization, whereas receipt of ≥1 dose was negatively associated with hospitalization. **Conclusions:** Infants ≤2 months are at high risk for severe pertussis and hospitalization. Early diagnosis, timely vaccination, and close monitoring of children presenting with cyanosis, paroxysmal cough, and fever are critical to reducing pertussis-related hospitalization risk, especially in infants.

## 1. Introduction

Pertussis, which arises from *Bordetella pertussis* infection, is a respiratory infectious disease amenable to vaccine prevention, characterized by a protracted clinical course lasting several months [1]. In the pre-vaccine era, pertussis represented a primary source of morbidity and mortality in infants and preschool-aged children worldwide. Since diphtheria–tetanus–pertussis (DTP) combination vaccines were rolled out worldwide in 1974, the incidence of the disease dropped dramatically. Nevertheless, frequent pertussis recurrences have been widely documented over the past decade across nations maintaining high vaccination coverage [2,3], particularly in Europe and North American nations. The Global Pertussis Initiative (GPI), founded in 2001 [4], was established to systematically assess and prioritize diverse immunization approaches, such as routine childhood immunizations, universal booster vaccination for adolescents, adults, and preschoolers [5]. By issuing region-specific recommendations grounded in local epidemiology, the GPI also aims to enhance diagnostic capacity, surveillance, and vaccine uptake [6]. Regarding vaccine formulations, the Advisory Committee on Immunization Practices (ACIP) recommends diphtheria, tetanus toxoid, and acellular pertussis (DTaP) for children <7 years and tetanus toxoid, reduced diphtheria toxoid, and acellular pertussis (Tdap) for adolescents and adults. A single dose of Tdap is advised for non-pregnant individuals aged ≥11 years. Additionally, ACIP recommends that pregnant women receive Tdap during 27–36 weeks of gestation and that adults receive booster doses of tetanus and diphtheria toxoid (Td) vaccine every 10 years following one dose of Tdap [7].

Humans are the exclusive natural host of *Bordetella pertussis*, which could infect individuals across all age demographics. Shifts in disease transmission dynamics have altered the epidemiological landscape, with a rising proportion of infections currently. In the United States, more than half of cases occurred in adolescents and adults during the 1990s [8], and by 2015 the highest proportion of cases was among adolescents aged 11–19 years [9]. China released the national Diagnosis and Treatment Protocol for Pertussis (2023 Edition) in December 2023 [10]. Following the nationwide implementation of this guideline, 494,321 cases were recorded in 2024 [11], a sharp increase from 38,295 cases in 2022 [12], all provinces reporting substantially higher numbers than in previous years [13]. Jiangsu Province was also severely affected during this period.

Children aged 0–6 years are susceptible to pertussis owing to immature immunity. They are prone to complications including pneumonia and encephalopathy after infection [14] with poor prognosis, making this age group a key population of concern. Although pertussis in this age group may present with certain characteristic clinical features, infants and young children are frequently underdiagnosed or misdiagnosed owing to atypical manifestations or the absence of the classic inspiratory “whoop”. Moreover, the close interactions among this age group within households and daycare settings readily facilitate cluster transmission [14], thus perpetuating the ongoing epidemic. Nevertheless, despite some regional investigations having been conducted by researchers [15,16], robust epidemiological data and clinical characteristics for children aged 0–6 years in Jiangsu Province remain insufficient.

Therefore, we performed a cross-sectional study collecting individual case data and clinical information from children aged 0–6 years with pertussis in Jiangsu Province during January–May 2024. By analyzing clinical characteristics and identifying risk factors associated with hospitalization, we aimed to provide scientific evidence supporting pertussis prevention and control strategies in Jiangsu.

## 2. Materials and Methods

### 2.1. Study Population and Data Sources

This cross-sectional study enrolled children with pertussis diagnosed in tertiary hospitals and children’s hospitals across Jiangsu Province between January and May 2024 in line with the Diagnosis and Treatment Protocol for Pertussis (2023 Edition) [10]. Clinical data were extracted from hospital electronic medical record systems, with missing core information supplemented via telephone follow-up. Vaccination histories were retrieved from Jiangsu Provincial Immunization Information System using children’s identity card numbers or a combination of full name plus birth date.

### 2.2. Case Definition

Suspected cases were defined as individuals with spasmodic cough lasting ≥2 weeks; or infants with a history of exposure to pertussis cases presenting with recurrent apnea, asphyxia, cyanosis, bradycardia, or intermittent paroxysmal cough; or older children, adolescents, or adults with a history of exposure to pertussis cases and unexplained afebrile cough >2 weeks.

Clinically diagnosed cases were defined as suspected cases presenting with elevated peripheral blood leukocyte and lymphocyte counts, or individuals with spasmodic cough lasting ≥2 weeks who had a clear epidemiological link to a confirmed pertussis case.

Confirmed cases were defined as suspected or clinically diagnosed cases who met any of the following laboratory criteria: (1) positive Bordetella pertussis culture; (2) positive nucleic acid testing; (3) seroconversion with a ≥4-fold rise in IgG antibody titers between acute and convalescent phases (individuals without pertussis vaccination or prior infection within the preceding year).

### 2.3. Inclusion and Exclusion Criteria

#### 2.3.1. Inclusion Criteria

(1) Aged 0–6 years; (2) permanent residency in Jiangsu Province; (3) consistent with the above diagnostic criteria for pertussis; (4) complete accessible clinical information and retrievable vaccination records.

#### 2.3.2. Exclusion Criteria

(1) Severe congenital disorders or concurrent acute infectious diseases that may confound pertussis diagnosis; (2) major missing clinical or immunization data; (3) duplicate case entries.

### 2.4. Quality Control

All investigators received standardized training prior to data collection. Periodic quality audits were implemented throughout data collection to identify entry or logical errors, which were sent back for timely revision. Prior to statistical analysis, the dataset was cleaned to remove outliers and guarantee data integrity and accuracy.

### 2.5. Statistical Analysis

Data were double-entered into an EpiData database before export to Microsoft Excel for analysis. Categorical variables were described by frequencies and percentages [*n* (%)], and intergroup differences were compared using the χ^2^ test or Fisher’s exact test, as appropriate. Logistic regression was performed to identify factors associated with hospitalization. Variables with *p* < 0.05 in univariate analysis were entered into a multivariable logistic regression model using a forward stepwise selection procedure with entry and removal criteria set at *p* < 0.05 and *p* > 0.10, respectively. Adjusted odds ratios (ORs) with 95% confidence intervals (CIs) were calculated. The discriminatory performance of the final model was assessed by the area under the receiver operating characteristic (ROC) curve (AUC). All statistical analyses were performed using R software (version 4.3.0), and a two-tailed *p* < 0.05 was considered statistically significant.

## 3. Results

### 3.1. Basic Characteristics of the Cases

Between January and May 2024, 3361 pertussis cases aged 0–6 years were included in this study. Of these, 51.4% were male (1726 cases), and 48.6% were female (1635 cases). The proportions of children aged 0–2 months, 3–5 months, 6–23 months, 2 years, 3 years, 4 years, 5 years, and 6 years were 5.9% (199 cases), 7.4% (249 cases), 9.1% (305 cases), 3.6% (120 cases), 5.4% (182 cases), 10.5% (354 cases), 22.6% (761 cases), and 35.4% (1191 cases), respectively. Overall, 78.0% of cases were reported in southern Jiangsu (2622 cases). Regarding pertussis vaccination status, 4.1% (139 cases) had received one dose, 1.5% (49 cases) two doses, 6.9% (231 cases) three doses, and 76.8% (2581 cases) four doses, whereas 10.7% (361 cases) remained unvaccinated due to contraindications, age ineligibility, or other reasons. Confirmed cases constituted 89.0% (2982 cases), while clinically diagnosed cases accounted for the remaining 11.0% (368 cases).

### 3.2. Clinical Characteristics

Cough was present in nearly all children (3259/3361), with 52.7% (1771/3361) of them exhibiting typical paroxysmal coughing. The proportions of children presenting with fever, abnormal breath sounds, vomiting, cyanosis, and apnea were 24.9% (828 cases), 12.7% (428 cases), 17.4% (585 cases), 3.1% (105 cases), and 1.2% (42 cases), respectively.

(1) Age Distribution

Cough occurred in up to 95% of children across all age groups, with no statistically significant difference between groups (*p* = 0.588). In contrast, paroxysmal cough and abnormal breath sounds were more frequent in children aged ≤2 years than in those aged 3–6 years, with the highest proportions observed in the 3–5 months age group. Cyanosis and apnea occurred predominantly in infants aged 0–23 months and decreased gradually with age. The highest rate of fever was observed in the 6–23 months age group (33.8%) and the lowest in the 0–2 months group (19.4%). The proportions of vomiting ranged from 12.7% to 27.9% across age groups. Cyanosis was most prevalent in the 3–5-month group (11.2%), whereas apnea reached its highest proportion in the 0–2-month cohort (6.0%). The prevalence of both symptoms decreased to ≤3.3% and ≤1.7%, respectively, among children aged ≥3 years (Table 1). Statistically significant differences across age groups were observed in all clinical symptoms except cough (*p* < 0.05).

(2) Vaccination Doses

Significant differences were identified in the proportions of fever, paroxysmal cough, abnormal breath sounds, vomiting, cyanosis, and apnea across different vaccination dos-es. Compared with children in other immunization categories, those who completed the 4-dose vaccination series had the lowest proportions of paroxysmal cough (50.3%), abnormal breath sounds (10.7%), and vomiting (15.8%) (Table 1). Table 1 also shows that vaccination significantly mitigated severe symptoms such as cyanosis and apnea, and that the proportions of these two symptoms progressively decreased with an increasing number of vaccine doses received.

(3) Time interval between symptom onset and diagnosis

Significant differences were observed across groups with different time intervals between symptom onset and diagnosis in the occurrence of cough, paroxysmal cough, expiratory breath sounds, vomiting, and cyanosis (*p* < 0.05). As presented in Table 2, the overall proportions of cough remained high, with the highest in the 15-30 days group (98.7%) and the lowest in the <7 days group (94.0%). The proportion of paroxysmal cough peaked at 7–14 days after onset (56.9%) and was lowest in the <7 days group (46.3%). The proportion of expiratory breath sounds was relatively similar across groups (12.5–13.1%). The proportion of vomiting showed a progressive increase with longer disease duration, being highest in the >30 days group (21.3%) and lowest in the <7 days group (13.7%). The proportion of cyanosis was also highest in the >30 days group (4.5%). No statistically significant difference was found in the proportions of apnea across groups (*p* = 0.211).

(4) Type of Medical Visit

**Figure 1:**
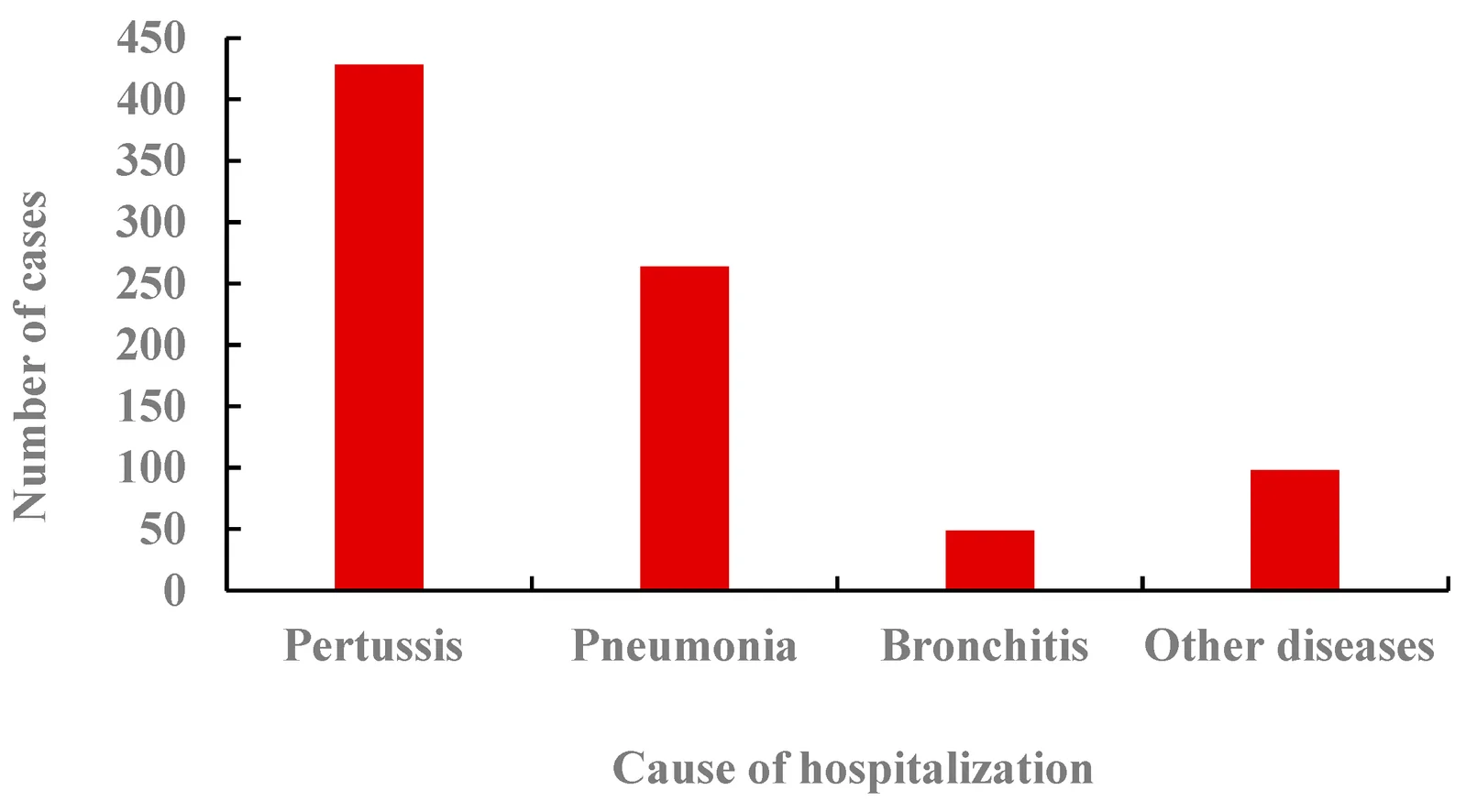
Distribution of causes of hospitalization among children aged 0–6 years with pertussis.

Based on medical visit records, hospitalized children accounted for 25.5% (856 cases) and outpatients for 74.5% (2505 cases). Cough was a common symptom in both inpatients and outpatients, but its proportion was higher in hospitalized children (98.6%) than in outpatients (96.4%). Moreover, hospitalized children had significantly higher proportions of paroxysmal cough (66.6% vs. 47.9%), expiratory breath sounds (17.2% vs. 11.2%), vomiting (21.0% vs. 16.2%), cyanosis (7.2% vs. 1.7%), and apnea (3.5% vs. 1.1%) compared with outpatients (Table 2). Further analysis of the reasons for hospitalization among the 856 hospitalized children revealed that 428 cases (50.00%) were directly hospitalized due to pertussis, 264 cases (30.8%) due to pneumonia, 49 cases (5.7%) due to bronchitis, and the remaining 115 cases (13.4%) due to other diseases (Figure 1).

### 3.3. Factors Associated with Hospitalization

**Figure 2:**
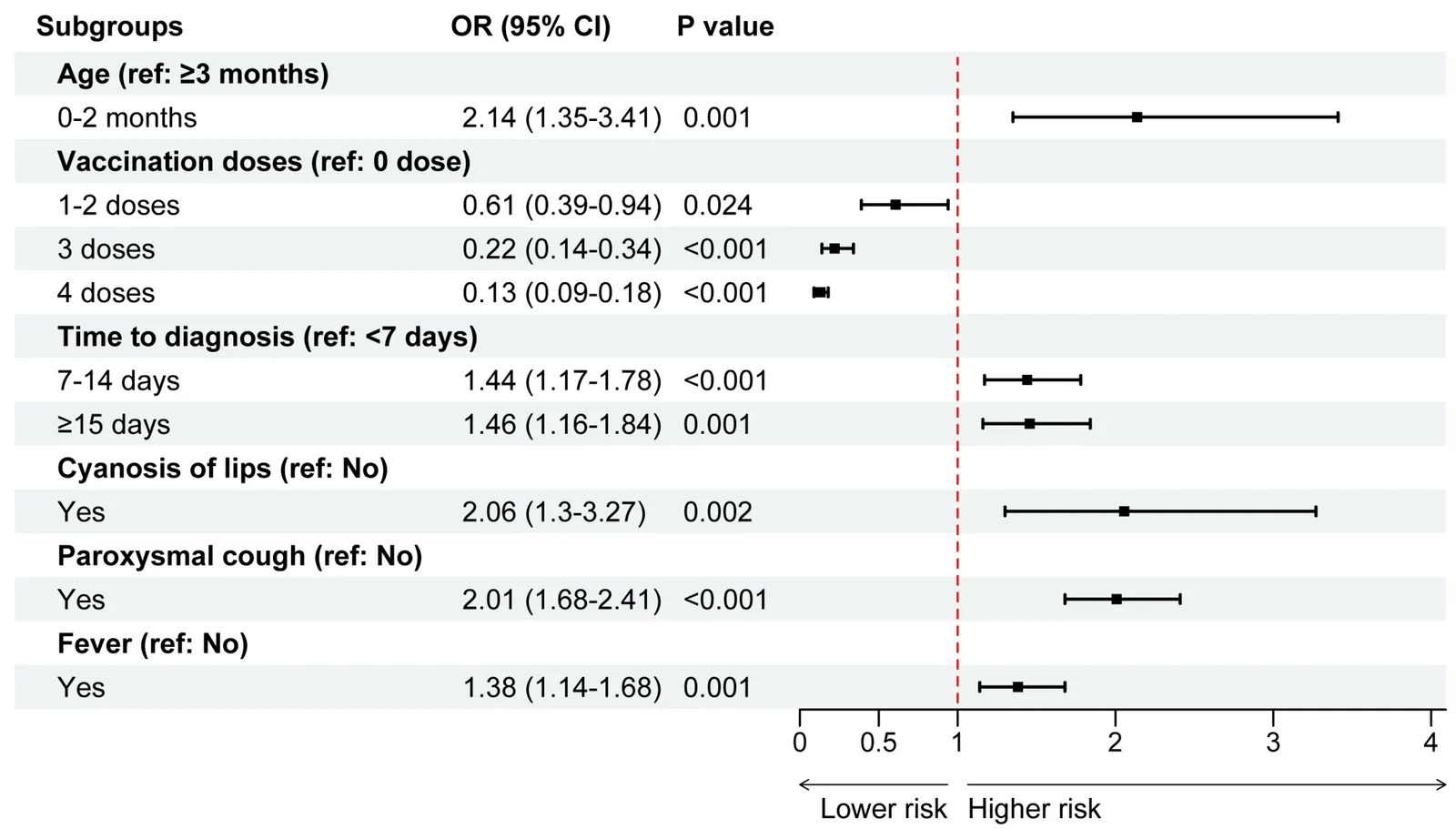
Forest plot of multivariate logistic regression analysis of factors associated with hospitalization in children aged 0–6 years with pertussis.

Univariate analysis, with hospitalization status as the dependent variable and age, sex, vaccination doses, time interval between symptom onset and diagnosis, fever, cough, paroxysmal cough, expiratory whoop, vomiting, cyanosis, and apnea as independent variables, demonstrated that all independent variables except sex were statistically significantly associated with hospitalization (*p* < 0.05). Multivariate logistic regression analysis identified that children aged 0–2 months were positively correlated with hospitalization (OR = 2.14, 95% CI: 1.35–3.41). Compared with the 0-dose vaccination group, children who received 1–2 doses (OR = 0.61, 95% CI: 0.39–0.94), 3 doses (OR = 0.22, 95% CI: 0.14–0.34), or 4 doses (OR = 0.13, 95% CI: 0.09–0.18) had significantly lower odds of hospitalization. Compared with those diagnosed within <7 days from onset, children diagnosed at 7–14 days (OR = 1.44, 95% CI: 1.17–1.78) or ≥15 days (OR = 1.46, 95% CI: 1.16–1.84) had significantly higher odds of hospitalization. The presence of cyanosis (OR = 2.06, 95% CI: 1.30–3.27), paroxysmal cough (OR = 2.01, 95% CI: 1.68–2.41), and fever (OR = 1.38, 95% CI: 1.14–1.68) was significantly associated with hospitalization (Figure 2). The area under the ROC curve (AUC) was 0.748 (95% CI: 0.728–0.768), indicating that the model had good discriminative ability for distinguishing hospitalized children from outpatients.

## 4. Discussion

This study, based on clinical and immunization data from 3361 children aged 0-6 years with pertussis in Jiangsu Province from January to May 2024, found that hospitalization was independently positively associated with young age (0–2 months), delayed diagnosis (diagnosed ≥7 days), incomplete vaccination, and the presence of cyanosis, paroxysmal cough, and fever. These results underscore the importance of early diagnosis and timely vaccination to reduce hospitalization burden in pediatric pertussis.

Children aged six years accounted for the largest proportion of enrolled cases at 35.4%, whereas infants aged 0–2 months made up only 5.9%. The observed age shifts differed substantially from national surveillance data (2011–2017) [17] and Jiangsu provincial surveillance data (2005–2016) [18], which may be related to waning vaccine-induced antibodies. Currently, preschool children in China receive DTaP vaccines, which have a better safety profile than whole-cell pertussis vaccines. However, DTaP offers limited long-term seroprotection, and antibody titers may fall to 0–20% of peak levels within five years post-vaccination [19,20], leaving preschool children vulnerable to pertussis infection. To address immunity gaps resulting from progressive antibody waning, a revised national pertussis immunization schedule was implemented in 2025, which adds a DTaP booster dose at six years of age to protect school-aged children [21]. In addition, advances in laboratory diagnostic approaches such as PCR have improved identification of children with atypical clinical manifestations, leading to a marked rise in confirmed pertussis cases compared with earlier periods. Geographically, 78.0% of all enrolled cases were concentrated in southern Jiangsu, which may be attributable to the region’s abundant healthcare resources, higher healthcare-seeking awareness among caregivers, and elevated population density [22].

Although classic pertussis manifestations, including inspiratory whoop and paroxysmal cough, can be observed, most patients present with non-specific mild symptoms at clinical consultation [23], with relatively mild coughing spells. Cough is nearly universal across all age subgroups at comparable frequencies, consistent with previous epidemiological findings [24]. Multiple prior studies have validated that severe complications such as cyanosis and apnea predominate among infants [12], and disease severity inversely correlates with age; younger infants tend to develop more severe illness and have higher odds of hospitalization. Multivariate regression revealed that infants aged 0–2 months had a 2.14-fold higher hospitalization risk relative to children ≥3 months old, aligning with published data from Zhejiang [16] and Beijing [25]. This disparity may stem from two factors: first, infants aged 0–2 months have not completed primary immunization and possess inadequate maternally derived antibodies for effective protection [26]; second, infants feature narrow airway lumens, impaired cough clearance and immature respiratory tracts, predisposing to retained secretions, airway obstruction and apnea episodes [16]. Our study further revealed a statistically significant positive association between cyanosis, fever, and paroxysmal cough and the risk of hospitalization. This finding is clinically plausible, as hospitalized children generally present with greater illness severity than outpatients. Consistent with our observations, a study from Beijing that enrolled 967 children with pertussis reported significantly higher proportions of cyanosis, fever, and paroxysmal cough in the severe disease group than in the non-severe group [24].

In our study, children receiving four-dose DTaP vaccine demonstrated the lowest prevalence of all symptoms except cough, whereby the proportion of critical outcomes including cyanosis and apnea declined progressively with increasing vaccine doses. A study from Oregon, USA, which included 624 pertussis cases aged 6 weeks to 18 years, found that pertussis vaccination effectively alleviates illness severity, shortens disease duration, and reduces the incidence of severe disease [27]. Likewise, a study conducted in Scotland showed that full vaccination against pertussis was associated with a 69% reduction in hospitalization rates compared with non-vaccination among children [28]. Children in our study who received ≥1 dose of DTaP vaccine exhibited markedly lower odds of hospitalization than unvaccinated children. These findings clearly demonstrate that pertussis vaccination is associated with both decreased pediatric susceptibility to infection and mitigation of clinical severity and hospitalization probability among breakthrough cases [29].

Pertussis has an incubation period of 5–21 days. Currently, pertussis cases often present with atypical symptoms, and limited clinical awareness among some clinicians delays diagnostic suspicion until children have experienced ≥14 consecutive days of cough, leading to lost optimal treatment opportunities and unfavorable disease progression. Our study found that 26.89% of pediatric patients were diagnosed more than 15 days after symptom onset. These patients had markedly higher rates of vomiting and cyanosis of the lips, which is consistent with the findings reported by Bin et al. [30]. A review conducted by Xu et al. [31] indicated that delayed diagnosis resulting from failure to identify atypical cases in a timely manner can exacerbate pertussis and increase the risk of severe illness. Our analysis demonstrated that a diagnostic delay of ≥7 days was positively associated with hospitalization. Accordingly, timely identification, diagnosis, and treatment are clinically crucial for mitigating symptoms and cutting pediatric hospitalization rates.

Our study has several potential limitations that should be considered. First, given that all clinical data were retrospectively abstracted from electronic medical records and partial missing information supplemented by telephone interviews with caregivers, information bias and recall bias could be introduced in the present study. Second, all patients were recruited from tertiary and specialized children’s hospitals, while cases reported from primary and secondary healthcare, as well as asymptomatic carriers, were missing. This may introduce selection bias and lead to an underestimation of the true burden of pertussis. Third, the study did not evaluate the impacts of antimicrobial resistance of *Bordetella* isolates and personalized treatment strategies on pediatric in-hospital outcomes, which represents a direction for future research.

## 5. Conclusions

The ongoing pertussis epidemic among children in Jiangsu predominantly affects older preschool-aged youngsters, whereas young infants remain at the highest risk of severe disease. Complete 4-dose DTaP vaccination substantially alleviates clinical manifestations and reduces the odds of hospitalization. In contrast, delayed diagnosis is associated with greater disease severity and increased admission odds. Accordingly, strategies to ensure vaccination compliance, implement a 6-year booster, improve clinician training on atypical pertussis recognition, and closely monitor children with cyanosis, paroxysmal cough, and fever are warranted to optimize provincial prevention and control efforts.

## Figures and Tables

**Table 1:** Comparison of clinical characteristics among children of different age groups and vaccination doses [*n* (%)].

Group	Cases (*n* = 3361)	Fever (*n* = 828)	Cough (*n* = 3259)	Paroxysmal Cough (*n* = 1771)	Expiratory Breath Sounds (*n* = 428)	Vomiting (*n* = 585)	Cyanosis (*n* = 105)	Apnea (*n* = 58)
**Age**								
0–2 months	199	38 (19.4)	192 (96.5)	118 (59.3)	33 (16.6)	29 (14.6)	20 (10.1)	12 (6.0)
3-5 months	249	60 (24.3)	247 (99.2)	160 (64.3)	64 (25.7)	54 (21.7)	28 (11.2)	8 (3.2)
6-23 months	305	102 (33.8)	296 (97.0)	166 (54.4)	49 (16.1)	85 (27.9)	14 (4.6)	15 (4.9)
2 years old	120	30 (25.4)	117 (97.5)	69 (57.5)	21 (17.5)	31 (25.8)	2 (1.7)	2 (1.7)
3 years old	182	49 (27.2)	174 (95.6)	90 (49.5)	14 (7.7)	33 (18.1)	6 (3.3)	1 (0.5)
4 years old	354	104 (29.9)	342 (96.6)	172 (48.6)	35 (9.9)	45 (12.7)	9 (2.5)	5 (1.4)
5 years old	761	175 (23.2)	739 (97.1)	380 (49.9)	79 (10.4)	122 (16.0)	15 (2.0)	5 (0.7)
6 years old	1191	270 (22.9)	1152 (96.7)	616 (51.7)	133 (11.2)	186 (15.6)	11 (0.9)	10 (0.8)
*p*-value		0.001	0.588	0.004	<0.001	<0.001	<0.001	<0.001
**Vaccination doses**								
0 doses	361	85 (23.8)	353 (97.8)	227 (62.9)	74 (20.5)	63 (17.5)	36 (10.0)	18 (5.0)
1–2 doses	188	47 (25.3)	186 (98.9)	119 (63.3)	45 (23.9)	50 (26.6)	20 (10.6)	9 (4.8)
3 doses	231	75 (32.8)	223 (96.5)	126 (54.5)	33 (14.3)	64 (27.7)	8 (3.5)	8 (3.5)
4 doses	2581	621 (24.4)	2497 (96.7)	1299 (50.3)	276 (10.7)	408 (15.8)	41 (1.6)	23 (0.9)
*p-value*		0.043	0.114	<0.001	<0.001	<0.001	<0.001	<0.001

**Table 2:** Comparison of clinical characteristics among children of different time intervals between symptom onset and diagnosis and type of hospital visit [*n* (%)].

Group	Cases (*n* = 3361)	Fever (*n* = 828)	Cough (*n* = 3259)	Paroxysmal Cough (*n* = 1771)	Expiratory Breath Sounds (*n* = 428)	Vomiting (*n* = 585)	Cyanosis (*n* = 105)	Apnea (*n* =58)
**Time interval between symptom onset and diagnosis**								
<7days	1092	295 (27.3)	1027 (94.0)	506 (46.3)	136 (12.5)	150 (13.7)	32 (2.9)	14 (1.3)
7-14days	1365	317 (23.5)	1344 (98.5)	777 (56.9)	174 (12.7)	249 (18.2)	44 (3.2)	21 (1.5)
15-29days	749	165 (22.2)	739 (98.7)	409 (54.6)	98 (13.1)	153 (20.4)	22 (2.9)	19 (2.5)
≥30 days	155	51 (33.8)	149 (96.1)	79 (51.0)	20 (12.9)	33 (21.3)	7 (4.5)	4 (2.6)
*p-value*		0.003	<0.001	<0.001	0.015	0.008	0.039	0.211
**Type of hospital visit**								
Inpatient	856	239 (28.3)	844 (98.6)	570 (66.6)	147 (17.2)	180 (21.0)	62 (7.2)	30 (3.5)
Outpatient	2505	589 (23.8)	2415 (96.4)	1201 (47.9)	281 (11.2)	405 (16.2)	43 (1.7)	28 (1.1)
*p*-*value*		0.01	0.004	<0.001	<0.001	0.002	<0.001	<0.001

## Data Availability

The datasets supporting the findings of this study are not publicly available owing to restrictions related to participant privacy and institutional ethical approval.
